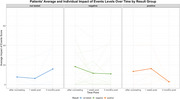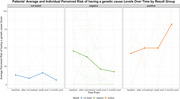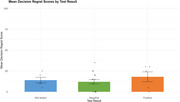# Impact of Genetic Testing for Familial Dementia: Experiences of Patients and families

**DOI:** 10.1002/alz70858_097600

**Published:** 2025-12-24

**Authors:** Jetske van der Schaar, Leonie N.C. Visser, Eva Asscher, Yolande A.L. Pijnenburg, Christa de Geus, Wiesje M. van der Flier, Annelien L. Bredenoord, Mariette A. van den Hoven, Sven J van der Lee

**Affiliations:** ^1^ Alzheimer Center Amsterdam, Neurology, Vrije Universiteit Amsterdam, Amsterdam UMC location VUmc, Amsterdam, Netherlands; ^2^ Amsterdam Neuroscience, Neurodegeneration, Amsterdam, Netherlands; ^3^ Section Genomics of Neurodegenerative Diseases and Aging, Department of Human Genetics, Vrije Universiteit Amsterdam, Amsterdam, Noord‐Holland, Netherlands; ^4^ Medical Psychology, Amsterdam UMC location AMC, University of Amsterdam, Amsterdam, Netherlands; ^5^ Department of Bioethics and Health Humanities, Julius Center for Health Sciences and Primary Care, University Medical Center Utrecht, Utrecht University, Utrecht, Utrecht, Netherlands; ^6^ Amsterdam Public Health, Quality of Care, Amsterdam, Netherlands; ^7^ Department of Ethics, Law and Humanities, Amsterdam UMC, Amsterdam, Noord‐Holland, Netherlands; ^8^ Clinical Genetics, Human Genetics, Vrije Universiteit Amsterdam, Amsterdam UMC location VUmc, Amsterdam, The Netherlands, Amsterdam, Netherlands; ^9^ Department of Epidemiology and Data Science, Vrije Universiteit Amsterdam, Amsterdam UMC, Amsterdam, North Holland, Netherlands; ^10^ Alzheimer Center, Department of Neurology, Amsterdam UMC, Vrije Universiteit Amsterdam, Amsterdam Neuroscience, Amsterdam, Netherlands; ^11^ Erasmus School of Philosophy, Erasmus University Rotterdam, Rotterdam, Zuid‐Holland, Netherlands

## Abstract

**Background:**

Memory clinic patients are interested in genetic testing for monogenic causes of dementia, but it remains unknown how they experience receiving the results. We explored the psychosocial and behavioral impact of DNA diagnostics on patients and their families.

**Methods:**

In this mixed‐methods study, 37 patients visiting Alzheimer Center Amsterdam, meeting eligibility criteria, were offered genetic testing for monogenic causes as part of their diagnostic work‐up. Patients were 41% female and aged 61±7 years (MMSE=22±5, 1 Subjective Cognitive Decline, 1 MCI, 30 dementia [20 AD, 4 PPA, 6 other], 5 other/undetermined). Six declined testing, 25 tested negative and six positive. Per potential proband, we included patients (*n* = 4), one or two relatives (*n* = 4), or both (*n* = 29). Relatives (*n* = 34) were 59% female, and aged 54±13. Participants completed questionnaires assessing psychosocial and behavioral factors (including anxiety, depression and distress) at first visit, one week after counseling, one week and three months after disclosure. We used linear mixed models to calculate effects of group (un‐tested, negative, or positive), time, and interactions on outcome variables, with patients and relatives analyzed separately. In addition, 9 patients and 11 relatives participated in 14 semi‐structured interviews. Verbatim transcripts were analyzed inductively.

**Results:**

Average anxiety, depression and distress levels remained below clinical threshold and did not change between groups or over time, in patients nor relatives (see figures). Three months after disclosure, patients tested positive showed an increase in perceived risk of carrying a monogenic cause (β=48.55, 95%CI[9.17,87.93], *p* <0.05). In addition, patients tested positive expressed greater willingness to participate in research than those un‐tested (scale:1‐5, 4.6vs2.6, *p* <0.05). Decision‐regret was low and independent of group (scale:1‐100, 13.6, *p* >0.05). Interviews revealed patients tested negative were relieved, reassured their offspring were not at genetic risk, but sometimes unsatisfied for not knowing the cause of their disease. Those tested positive appreciated having clarity, yet experienced emotional distress about potential implications for their children.

**Conclusions:**

Genetic testing for monogenic causes of dementia did not cause short‐term psychosocial harm. Patients understood the gist of the results, deemed them actionable and reported no regret. Further research in larger populations should confirm and expand upon these findings.